# A Motion Planner Based on Mask-D3QN of Quadruped Robot Motion for Steam Generator

**DOI:** 10.3390/biomimetics9100592

**Published:** 2024-09-30

**Authors:** Biying Xu, Xuehe Zhang, Xuan Yu, Yue Ou, Kuan Zhang, Hegao Cai, Jie Zhao, Jizhuang Fan

**Affiliations:** State Key Laboratory of Robotics and System, Harbin Institute of Technology, Harbin 150001, China; xubiying1108@163.com (B.X.); cosin_yv@163.com (X.Y.); yue.ou@hit.edu.cn (Y.O.); zkuan1996@foxmail.com (K.Z.); hgcai@hope.hit.edu.cn (H.C.); jzhao@hit.edu.cn (J.Z.)

**Keywords:** crawling robot, deep reinforcement learning, motion planning, hierarchical planning

## Abstract

Crawling robots are the focus of intelligent inspection research, and the main feature of this type of robot is the flexibility of in-plane attitude adjustment. The crawling robot HIT_Spibot is a new type of steam generator heat transfer tube inspection robot with a unique mobility capability different from traditional quadrupedal robots. This paper introduces a hierarchical motion planning approach for HIT_Spibot, aiming to achieve efficient and agile maneuverability. The proposed method integrates three distinct planners to handle complex motion tasks: a nonlinear optimization-based base motion planner, a TOPSIS-based base orientation planner, and a Mask-D3QN (MD3QN) algorithm-based gait motion planner. Initially, the robot’s base and foot workspace were delineated through envelope analysis, followed by trajectory computation using Larangian methods. Subsequently, the TOPSIS algorithm was employed to establish an evaluation framework conducive to foundational turning planning. Finally, the MD3QN algorithm trained foot-points to facilitate robot movement along predefined paths. Experimental results demonstrated the method’s adaptability across diverse tube structures, showcasing robust performance even in environments with random obstacles. Compared to the D3QN algorithm, MD3QN achieved a 100% success rate, enhanced average overall scores by 6.27%, reduced average stride lengths by 39.04%, and attained a stability rate of 58.02%. These results not only validate the effectiveness and practicality of the method but also showcase the significant potential of HIT_Spibot in the field of industrial inspection.

## 1. Introduction

With the rapid development of nuclear energy, research on robots in the nuclear power field has become increasingly prevalent [1]. Within pressurized water reactors of nuclear power plants, the heat transfer tubes within steam generators (SG) serve as critical components for heat exchange, which require regular inspection and maintenance. However, the specifications of tubes within steam generators vary, as do the distribution of the tube plates. There are many kinds of inspection robots in service, such as ZR-100 [2], PEGASYS [3], HIT-Crawler [4], etc. However, each robot can only be applied to a specific type of steam generator, featuring limited motion capabilities. Consequently, inspection tasks for different steam generators necessitate the corresponding robots, followed by re-installment and re-calibration, leading to suboptimal inspection efficiency.

To enhance the adaptability of robots to various types and specifications of tube plates, enabling them to move and inspect tube holes on the plate more flexibly, safely, and efficiently, we have developed a tube plate quadruped inspection robot named HIT_Spibot [5]. Concurrently, the development of a motion planner is required to ensure its rapid and reliable movement along specified paths on the tube plate.

Research on the locomotion of legged robots primarily focuses on ensuring stability, as these robots rely on contact forces such as friction to traverse on the ground. Ensuring stability is particularly crucial to prevent falls or overturning [6]. However, the toes of HIT_Spibot are firmly attached to the tube plate, allowing the robot to remain stable on the plate as long as three feet are fixed. Due to the unique character of the plate and robot structure, the objective of motion planning for tube plate quadruped inspection robots is to achieve flexible and efficient motion planning while ensuring safety. It is noteworthy that, to date, there has been no scholarly investigation into the motion planning of such robots engaged in complex planar movements.

**Figure 1:**
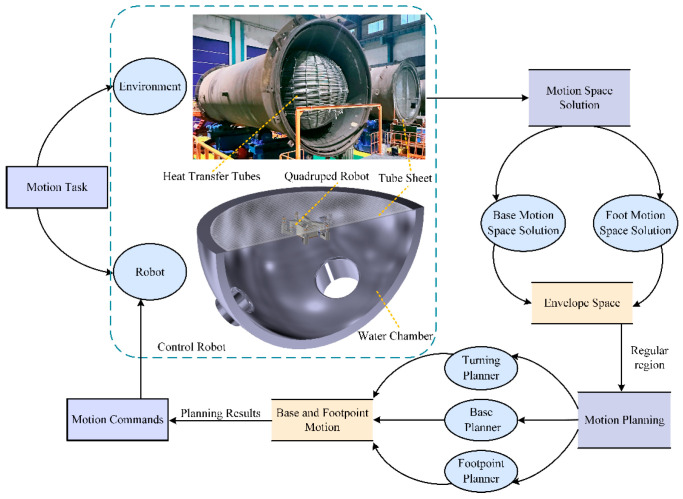
The motion planning system diagram of HIT_Spibot.

Therefore, this paper proposes a hierarchical planning method for the tube plate quadruped inspection robot. Utilizing an environment map constructed based on grid-based methods, the method designs the robot base motion planner, the robot base turning planner, and the gait motion planner, to enable the robot to move on the tube plate efficiently and safely. The task analysis and algorithm implementation framework are illustrated in Figure 1.

This paper aims to address three key issues: how to enhance planning efficiency using hierarchical planning, how to plan the motion of the robot’s base and foot-points, and how to utilize the results of hierarchical planning to realize robot motion. In this work, we make three significant contributions:

(1) We devised a base motion planner, formulating the base trajectory planning problem as a constrained nonlinear optimization problem. By employing the Lagrangian method, we attained the desired optimal solution to facilitate motion planning for the robot’s base.

(2) We established a base turning planner based on the TOPSIS method, enabling the robot to turn to an optimal initial pose, facilitating movement along the specified direction.

(3) We proposed a gait motion planner based on the MD3QN algorithm, designing a mask during the action selection and designing a reward function, thereby enabling autonomous learning of the robot foot-points. By integrating the outcomes of the above two planners, the robot can move on the tube plate flexibly and efficiently along the desired trajectory. Experimental validation demonstrates the effectiveness and advancement of the MD3QN algorithm.

The chapter arrangement of the paper is as follows. Section 2 presents the related work, and Section 3 introduces the structure of the robot, analyzing its motion characteristics and kinematics. Section 4 outlines the proposed algorithm. Section 5 provides experimental validation, and finally, the conclusion is presented.

## 2. Related Work

Early scholars typically employed model-based approaches for the motion planning of quadruped robots. The leg motion problem was transformed into a mathematical optimization problem, generating motion plans for quadruped robots facing complex terrain and tasks [7]. While this mathematical modeling approach is simple and feasible, it necessitates the establishment of sufficiently accurate models to compute a single trajectory optimization formula for leg motion, thereby determining the robot’s gait sequence and foot placement [8].

Although model-based methods can plan robot motion for various environments [9], each environment requires a separate analysis for its motion conditions, leading to substantial computation. In addition, traditional model-based planning methods in specific environments require exhaustive analyses of the robot’s motion characteristics, operating conditions, and potential future scenarios. Inadequate analyses may result in unsolvable motion planning.

With the gradual advancement of computer technology, scholars have discovered a more generalized and automated approach to achieve motion planning for quadruped robots. This involves specifying a high-level task, wherein algorithms determine the robot’s motion while considering the physical constraints of both foot and base movements. Given that the problem is properly formulated, ideally, the algorithm can generate motion for any high-level task and address motion planning issues at a generic level [7]. This is the planning method based on reinforcement learning (RL), where the control of robot motion strategies can be automatically generated through network learning. Moreover, the same learning method enables robots to learn optimal strategies across various environments [10].

On the other hand, as research on deep learning continues to deepen, deep reinforcement learning (DRL), combining deep learning with reinforcement learning, has been produced [11]. Researchers have found that applying DRL algorithms to motion planning for quadruped robots can prompt robots to adapt actively to the environments [12]. The essence of DRL algorithms lies in the agent learning which actions to take in a given environment to maximize numerical reward signals [13]. Based on the different actions output by robot planning, DRL algorithms can be categorized into methods based on value functions to address discrete action spaces, and methods based on policy gradients to address continuous action spaces [14].

Learning problems in continuous action spaces are more complex than those in discrete action spaces [15]. As the heat transfer tubes are distributed on specified tube plates, the motion planning can be transformed into planning discrete foot-points that meet task requirements. Compared to planning continuous joint angles, this method is more precise and straightforward, and it can also reduce unnecessary search space.

The earliest proposed DRL algorithm for solving discrete action space problems is the Deep Q-Network (DQN) algorithm [16]. This algorithm utilizes convolutional neural networks to approximate the state–action value function and reduces data correlation through the experience replay mechanism to improve learning efficiency. Simultaneously, it employs target Q-networks for training, providing reliable target values during temporal difference backups [17]. The application of the DQN algorithm to legged robots has also yielded significant results. Researchers constructed a discrete gait model for a hexapod robot and designed a method based on the DQN algorithm, planning a free gait with a high stability margin and a strong adaptability [18].

However, traditional DQN algorithms suffer from issues such as sparse rewards, low sample utilization, and overestimation. Therefore, many variants of DQN have been proposed. Among them, Double DQN primarily addresses the problem of overestimation by using different value functions to select actions and evaluate them [16]. On the other hand, Dueling DQN decomposes the output of the Q-values in the neural network into two parts: state value V and the difference A between action value and state value [19].

On the other hand, for the traditional DQN algorithm, Double DQN algorithm, Dueling DQN algorithm, D3QN algorithm, and other discrete space reinforcement learning algorithms, when there are infeasible actions in some environmental states, these RL algorithms do not screen the infeasible action space in specific circumstances when outputting action probability or score. As a result, a large number of early termination failures may occur in the training process, resulting in a decrease in the effectiveness of such training.

In task-oriented DRL, convergence difficulties inherent in RL itself render learning from scratch impractical for complex tasks. Therefore, hierarchically processing the required network structure and learning process is an intuitive choice. Jain et al. [20] proposed a hierarchical RL scheme enabling the Ghost robot to navigate along specific trajectories. This method employed a high-level policy network to control a low-level policy network, enabling the former to focus on determining the robot’s motion direction without learning the motor control commands. The authors of [21] introduced a hierarchical control method that employed hierarchical reinforcement learning to decompose complex tasks and yielded significant results in practice.

In summary, inspired by the advantages of hierarchical RL, we proposed a hierarchical motion planning method based on the MD3QN algorithm. This method comprised three layers: the base motion planner, the base turning planner, and the gait motion planner. Specifically, the gait motion planner, based on the MD3QN algorithm, was utilized to plan the robot foot-points, thereby achieving efficient, safe, and flexible robot motion.

## 3. Preliminaries

### 3.1. Robot Structure

**Figure 2:**
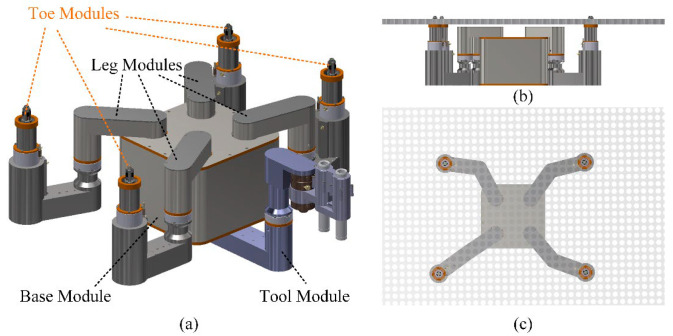
The structure of HIT_Spibot. (**a**) The motion modules; (**b**,**c**) denote the robot with four supporting feet.

The structure of the tube plate quadruped inspection robot HIT_Spibot is illustrated in Figure 2a. The robot consisted of a base module, four leg modules, four toe modules, and a tool module. Among them, there were two rotating joints on each leg module, and the relative rotation and combined motion between the leg module and the base module realized the robot walking on the tube plate. There were also two rotating joints on the tool module, and the relative rotation between the tool and the base modules positioned the eddy current tool precisely to the inspected heat transfer tubes. The design of the toe modules ensured that when three or four toes were fixed on the tube plate, the robot could be stably and reliably fixed to the tube plate. (The subsequent description and illustration hide the tool module, and the movement of the tool module was planned according to the movement of the leg modules, so that the tool movement would not interfere with the leg modules with no collision).

Through analysis of the robot’s structure, we could observe significant differences between the HIT_Spibot and traditional quadruped robots.

Stability Considerations: Traditional quadruped robots require sufficient stability margin during motion, commonly employing control methods such as Zero Moment Point [22] and Central Pattern Generators [23]. For other reptile-like quadruped robots, they always benefit from their larger supporting polygon to keep stable. For example, the turtle-inspired quadruped robot developed by Sun had more stable support polygons than other robots and could achieve stable walking turns and movements [24,25]. However, due to the structural specificity of the HIT_Spibot, as long as the toe modules were fixed to the tube plate, the robot could maintain stability without tipping over or falling. Nevertheless, considering the lifespan of components, it was advisable for the robot to minimize the stress on the toe modules during motion. This implies that the robot’s center of mass should be positioned as close as possible to the given base trajectory, preferably within the support polygon.

Foot-point Selection: Traditional quadruped robots need to consider terrain conditions, their own state, and the impact of accessibility on stability and speed. In contrast, for HIT_Spibot, any position satisfying the kinematic conditions could serve as a feasible foot-point.

### 3.2. Motion Analysis

**Figure 3:**
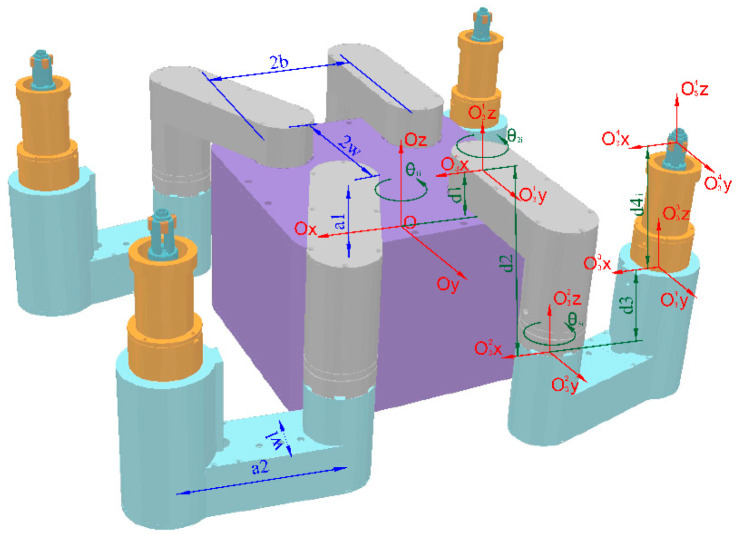
The coordinate system of HIT_Spibot.

The motion of HIT_Spibot can be categorized into the robot foot motion and base motion. Based on the robot’s configuration, we established the coordinate system as illustrated in Figure 3 and present the Denavit–Hartenberg parameters in Table 1.

**Figure 4:**
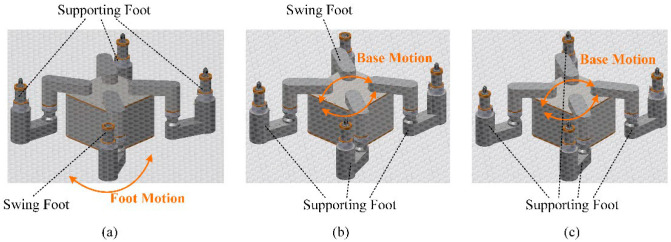
The motion of the robot base and foot. (**a**) 2R manipulator arm under three supporting feet; (**b**) 3-RRR parallel robot under three supporting feet; (**c**) 4-RRR parallel robot under four supporting feet.

The robot’s foot motion was as follows. Three legs acted as supporting feet, while the remaining one served as the swinging foot, forming a 2R-type serial mechanical arm with the robot base, as depicted in Figure 4a. Assuming the position of the robot base is denoted as $B(r_{B},\;c_{B})$, where $r_{B}$ and $c_{B}$ represent the row and column coordinates of the robot base, respectively, the forward kinematics simplified calculation formula for each foot $F_{i}(r_{i},\;c_{i})$ is as follows, where $\alpha_{i}=45+90\cdot (i\;-\;1),\;i\;=1,\;2,\;3,\;4$, denotes the angle of each foot’s rotation axis relative to the tube plate.

(1) $$\left\{\begin{array}{c} \begin{array}{c} r_{i}=r_{B}+\sqrt{b^{2}+w^{2}}\;\cos(\alpha_{i}+\theta_{1i})+{\;a}_{1}\;\cos(\alpha_{i}+\theta_{1i}+\theta_{2i})\;\\ +{\;a}_{2}\;\cos(\alpha_{i}+\theta_{1i}+\theta_{2i}+\theta_{3i}) \end{array}\\ \begin{array}{c} c_{i}=c_{B}+\sqrt{b^{2}+w^{2}}\;\sin(\alpha_{i}+\theta_{1i})+a_{1}\;\sin(\alpha_{i}+\theta_{1i}+\theta_{2i})\;\\ +a_{2}\;\sin(\alpha_{i}+\theta_{1i}+\theta_{2i}+\theta_{3i}) \end{array} \end{array}\right.$$

The robot base motion is driven by the contact forces between the feet and the tube plate. In the case of three or four supporting feet, the tube plate along with the fixed feet and robot base, we formed a 3-degree-of-freedom (DOF) parallel robot, as depicted in Figure 4b,c. Assuming the positions of the three supporting feet relative to the base are denoted as $F_{1}(r_{1},\;c_{1})$, $F_{2}(r_{2},\;c_{2})$, and $F_{3}(r_{3},\;c_{3})$, the simplified inverse kinematics formulas for the rotation angles $\theta_{2i}$ and $\theta_{3i}$ of each foot, are given as follows.

(2) $$\left\{\begin{array}{c} \theta_{2i}=(\arctan\frac{r_{i}}{c_{i}}\;\mathrm{or}\;\pm \frac{\pi }{2})\pm \arccos\frac{{r_{i}}^{2}+{c_{i}}^{2}+{a_{1}}^{2}-{a_{2}}^{2}}{2a_{1}\sqrt{{r_{i}}^{2}+{c_{i}}^{2}}}\\ \theta_{3i}=\arccos\frac{{r_{i}}^{2}+{c_{i}}^{2}-{{\;a}_{1}}^{2}-{\;a_{2}}^{2}}{2a_{1}a_{2}} \end{array}\right.$$

## 4. Method

Given the tube plate and the robot’s motion path, we considered the solved regular robot workspace as prior knowledge. Based on this, we planned the robot’s motion, including base motion, base turning, and gait motion.

### 4.1. Enveloping Method for the Workspace Solution

Due to the irregular workspace of the robot’s base and feet, exploration and computation were significant challenges. Therefore, this study considered envelope analysis to solve the regular workspace.

**Figure 5:**
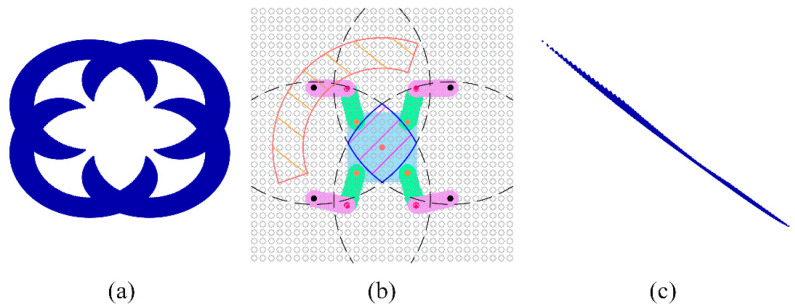
The workspace of the robot base and feet. (**a**) The robot feet workspace under the Monte Carlo method; (**b**) the boundaries of the workspace. The solid black circles represent the centroids of the circular arcs defining the base workspace, while the red circles represent the centroids of the circular arcs defining the feet’s motion space; (**c**) The robot base workspace under the Monte Carlo method.

According to the forward kinematic Equation (1) outlined in Section 3.2, a Monte Carlo method was employed to generate numerous random combinations of joint angles within the specified ranges for each leg of the robot while fixing the position and orientation of the robot’s base. The positions of the four feet were computed, and each corresponding configuration of joint angles was examined for potential self-collisions. Valid foot positions were recorded, leading to the delineation of the robot’s foot workspace. For a given base pose, the actual foot workspace is illustrated in Figure 5a, which is intersected by two lines, presenting a ring-shaped area as depicted in the orange-shaded region in Figure 5b. Similarly, using the inverse kinematics Equation (2) from Section 3.2 and the Monte Carlo method, a multitude of base positions were randomly generated within the quadrilateral formed by the four fixed foot positions. The joint angles of the robot were then calculated, checking for solutions and verifying whether these solutions fell within the respective motion spaces while also ensuring that no self-collisions occurred. Valid base positions were documented, resulting in the representation of the base’s workspace. For a specified robot feet position, the actual base workspace is depicted in Figure 5c, which is in the region formed by the intersection of four or three circular rings, as shown in the blue-shaded region in Figure 5b.

**Figure 6:**
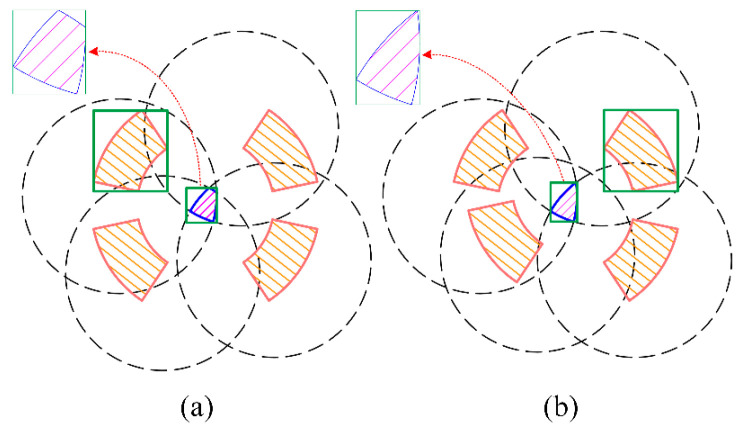
Workspace envelope rectangles. (**a**) The swing foot’s envelope rectangle and the base’s envelope rectangle under three supporting feet; (**b**) the swing foot’s envelope rectangle and the base’s envelope rectangle under four supporting feet.

We adopted minimum bounding rectangles to represent the workspaces of the robot base and feet. The foot workspace’s bounding area was necessarily a truncated ring. By considering the four endpoints of the truncated ring and the tangents of each circle, the foot workspace bounding rectangle could be determined. As for the base workspace, its bounding area was necessarily formed by the intersection points of three or four circles. By considering these intersection points and the tangents of each circle, the base workspace bounding rectangle could be determined, as illustrated in Figure 6.

### 4.2. Robot Base Motion Planner

Base motion planning involves determining feasible base trajectories when the foot swings. The points along the base trajectory should lie within the base workspace and align as closely as possible with the given path. Therefore, this section aimed to plan the local destinations for each base motion under three supporting feet and one swinging foot, modeling this process as a nonlinear optimization problem to obtain the optimal desired base position. For constrained optimization problems with inequality constraints, various solving methods can be employed. In this section, we employed the Lagrange multiplier method [26].

Assuming the starting point of the base is $P_{s}(x_{s},{\;y}_{s})$, the destination of the motion direction is $P_{e}(x_{e},\;y_{e})$, and the coordinates of the two corner points of the current supporting feet’s bounding rectangle are $R_{1}(a,\;c)$ and $R_{2}(b,\;d)$, the optimal desired base position $P_{b}(x,\;y)$ is formulated as follows:

(3) $$\left\{\begin{array}{c} \min\;f\left(x,\;y\right)={\left\|\vec{P_{s}P_{b}}\right\|}_{2}+{\left\|\vec{P_{e}P_{b}}\right\|}_{2}\\ \mathrm{subject}\;\mathrm{to}\;\begin{array}{c} g_{1}\left(x,\;y\right)=-x+\min(a,\;b)\leq 0\\ g_{2}\left(x,\;y\right)=x-\max(a,\;b)\leq 0\\ \begin{array}{c} g_{3}\left(x,\;y\right)=-y+\min(c,\;d)\leq 0\\ g_{4}\left(x,\;y\right)=y-\max(c,\;d)\leq 0 \end{array} \end{array} \end{array}\right.$$

where ${\left\|A\right\|}_{2}$ denotes the L2 norm of A, that is, ${\left\|\vec{P_{s}P_{b}}\right\|}_{2}=\frac{\left|x-x_{1}\right|}{\sqrt{{\left(x-x_{1}\right)}^{2}+{\left(y-y_{1}\right)}^{2}}}+\frac{\left|y-y_{1}\right|}{\sqrt{{\left(x-x_{1}\right)}^{2}+{\left(y-y_{1}\right)}^{2}}}$. Introducing Lagrange multipliers $\mu \{\mu_{i},\;i=1,\;\ldots ,\;4\}$, the corresponding Lagrangian function is formulated as follows:

(4) $$L(x,\;y,\;\mu )=f(x,\;y)+\sum_{j=1}^{n}\mu_{j}g_{j}\left(x,\;y\right)$$

By introducing the Karush–Kuhn–Tucker conditions as follows, the solution set for the optimal ideal base position can be obtained. The evaluation criterion is based on the distance from the global motion destination, where a closer distance indicates a more optimal base position, leading to the derivation of the optimal ideal base position $P_{b}(x^{*},\;y^{*})$.

(5) $$\begin{array}{c} \begin{array}{c} \nabla_{x,\;y}L(x,\;y,\;\mu )=0\\ g_{j}(x,\;y)\;\leq \;0,\;j=1,\;2,\;3,\;4 \end{array}\\ \mu_{j}\;\geq \;0\\ \mu_{j}g_{j}(x,\;y)=0 \end{array}$$

Near the position $P_{b}(x^{*},\;y^{*})$, we conducted a localized search to obtain an optimal feasible base position, satisfying the inverse kinematic feasible solutions. Through the process from the current base position to the optimal feasible base position, a series of base positions can be obtained by solving the inverse kinematics along the motion direction, thereby acquiring the local base motion trajectory.

### 4.3. Robot Base Turning Planner

**Figure 7:**
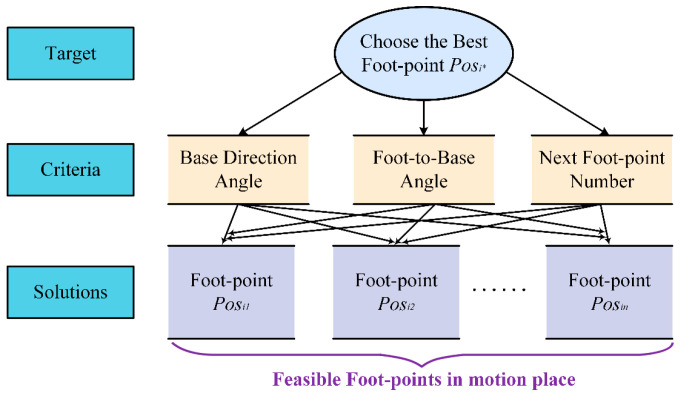
The evaluation system of the base turning motion. * represents the optimal solution.

By analyzing the robot’s motion characteristics and working environment, we can determine the desirable initial posture of the robot based on the following conditions: one of the robot’s orientations aligns as closely as possible with the given direction; the four feet are evenly distributed along this orientation to facilitate subsequent motion; and this posture ensures sufficient feasible space for the swinging foot to place at the start of motion, thereby enhancing the likelihood of movement. These conditions can be translated into evaluation criteria: Cost Index 1—the angle between the robot’s orientation and the given direction; Cost Index 2—the deviation of the foot angle relative to the base from the standard 45° angle; and Benefit Index 3—the size of the feasible set of foot-points for the next swinging foot. Therefore, the planning of the base turning planner can establish an evaluation system as depicted in Figure 7.

The objective of the base turning planning can be formulated as a Multi-Criteria Decision-Making (MCDM) problem. The most commonly used method for solving MCDM problems is the TOPSIS method, which is a multi-criteria approach that determines the optimal solution from a finite set of alternatives by minimizing the distance to the ideal point and maximizing the distance to the worst point [27]. The TOPSIS method is mathematically simple and offers flexible selection [28]. In this section, the base turning planning was based on the TOPSIS algorithm, and the process is outlined in Algorithm 1. Here, *n* represents the number of landing points to be evaluated, *i* denotes the landing point index, and the termination condition minimizes the angle between the orientation and the given direction while ensuring uniform distribution of the four feet.
**Algorithm 1** Turning Planning Based on TOPSISInput: target base angle $\alpha_{T}$ and current state *s*Output: Motion sequencesCompute feasible foot-point $I_{p},p\;=1,2,\cdots ,n$for p in range(*n*) do Compute base direction angle $x_{p1}$, foot to base angle $x_{p2}$ and next foot-point number $x_{p3}$endwhile not stopping condition(s) do Obtain TOPSIS score:  Step 1: Positivize Data  $x_{\mathrm{ij}}$ on Cost index index: xPQ^=max{xp}−xpq,q =1,2  $x_{\mathrm{ij}}$ on Benefit index index: $\hat{x_{\mathrm{PQ}}}=x_{\mathrm{pq}},q=3$  Step 2: Normalize Data  $z_{\mathrm{pq}}=\hat{x_{\mathrm{PQ}}}/\sqrt{\sum_{p=1}^{n}{x_{\mathrm{pq}}}^{2}}$
  Step 3: Determine the positive and negative ideal solutions  $z_{\mathrm{bt}\_p}=[\max\{x_{p1}\},\max\{x_{p2}\},\max\{x_{p3}\}],\;p\;=1,\;2,\cdots ,\;n$         $z_{\mathrm{wt}\_p}=[\min\{x_{p1}\},\min\{x_{p2}\},\min\{x_{p3}\}],\;p\;=1,\;2,\cdots ,\;n$  Step 4: Calculate the TOPSIS score       $S_{p}=\sum_{q=1}^{3}{\left(z_{\mathrm{wtq}\_p}-z_{\mathrm{pq}}\right)}^{2}/(\sum_{q=1}^{3}{\left(z_{\mathrm{wtq}\_p}-z_{\mathrm{pq}}\right)}^{2}+\sum_{q=1}^{3}{\left(z_{\mathrm{btq}\_p}-z_{\mathrm{pq}}\right)}^{2})$ Obtain foot-point ${I_{p}}^{*}=\max\{S_{p}\}$ Update state *s*

### 4.4. Robot Gait Motion Planner

Taking the robot posture output from the base turning planner as input to the gait motion planner, we start learning the robot’s motion along the given path from existing strategies, aiming to reduce the difficulty of DRL learning [18]. The network model trained by the D3QN algorithm obtained foot-points, then updated the status, repeating this process until reaching the destination. In this section, the robot’s free gait motion was to plan the foot-points, ensuring that the robot could reach the destination as quickly as possible and minimizing the force on the toe modules.

**Figure 8:**
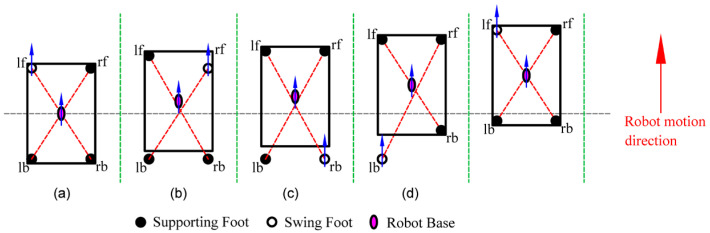
The foot-point sequence of HIT_Spibot. Arrows indicate the direction of motion for the feet or the base. (**a**) lf moves and base moves; (**b**) rf moves and base moves; (**c**) rb moves and base moves; (**d**) lb moves and base moves.

To enhance the flexibility of robot motion, this paper selected the foot-point sequence of the left front foot (lf), right front foot (rf), right back foot (rb), and left back foot (lb) along the robot motion direction, as illustrated in Figure 8.

The learning-based approach is that the agent learns by multiple interacting with the environment, encouraging positive behaviors and punishing negative ones. We addressed the sequential decision-making problem of interaction between an agent and its environment with the objective of maximizing the cumulative discounted reward. This problem was modeled as a discrete-time Markov Decision Process, consisting of a tuple ⟨S,A, P,R,γ⟩, where S represents the states of the robot, A represents actions, P represents the state transition distribution, R represents the reward function, and γ represents the discount factor.

During the training process, the state S served as the input to the robot’s motion planning policy network, while the action A served as the output of the policy network. The robot interacted with the environment based on the selected actions from the policy network, leading to transitions in the robot’s state. Simultaneously, the environment provided feedback through a reward function R. The policy network then selected the next action based on the updated state and reward function. The continuous updating of the policy network was the training process for the motion planner using RL algorithms.

Action Space A: For most quadruped robots employing RL, the action space typically consists of joint positions [29]. However, since each foot places on a specific hole on the plate, in order to reduce unnecessary exploration at the decision-making level, this paper defined the action space as a one-dimensional vector of discrete foot-point scores, as shown as follows. Each action corresponded to a foot-point $F_{k}(r_{F_{k}},\;c_{F_{k}})$, which was an offset relative to the current foot position $F_{c}(r_{F_{c}},\;c_{F_{c}})$, where $k=1,\;2,\;\cdots ,{\;k}_{\max}$. Here, ${\;k}_{\max}$ represents the number of foot-points that satisfy all possible inverse kinematics conditions and is also the dimension of the action space.

(6) $$a_{C}=[{\mathrm{Score}}_{1},{\;\mathrm{Score}}_{2},\;\cdots ,\;{\mathrm{Score}}_{k_{\max}}]$$

State Space S: The state space typically includes information such as the robot’s position and joint positions [21]. To simplify the learning process, this paper considered prior information on the accessibility of actions offline when designing the state space and added the target position to guide learning. The state space $s_{C}$ was defined as follows, containing the pose of the robot $P=[r_{B},c_{B},\theta_{1},\theta_{21},\;\theta_{31},\;\theta_{22},\;\theta_{32},\;\theta_{23},\theta_{33},\;\theta_{24},\;\theta_{34}]$, the global motion destination $T(r_{T},\;c_{T})$, and the judgment flag $f=[f_{1},\;f_{2},\;\cdots ,\;f_{k_{\max}}]$ indicating whether the foothold point corresponding to the action space can be landed on. Here, $f_{k}=1$ indicates that the foot-point is feasible, and $f_{k}=0$ indicates that the foot-point is not feasible.

(7) $$s_{C}=[P,{\;a}_{C},\;f,\;T]$$

Reward Function R: The reward function was set based on specific task objectives [29]. The reward function $R_{C}$ consisted of three parts and is designed as follows. One part was the regular penalty, where each step incurred a penalty $m_{0}$ to ensure that the robot did not move excessively or remain stationary; another part was the reward/penalty for forward/backward movement $R_{\mathrm{foot}}=[R_{\mathrm{foot}\_1},{\;R}_{\mathrm{foot}\_2},\;\cdots ,{\;R}_{\mathrm{foot}{\_k}_{\max}}]$; the third part was the stability reward/penalty $R_{\mathrm{stable}}=[R_{\mathrm{stable}\_1},\;R_{\mathrm{stable}\_2},\;\cdots ,{\;R}_{\mathrm{stable}{\_k}_{\max}}]$. Moreover, $l_{\mathrm{foot}\_k}$ represents the distance traveled by the foot along the motion direction, where a positive value indicates proximity to the target, a negative value indicates moving away from the target, and zero indicates stationary movement along the direction of motion; $l_{\mathrm{foot}\_\max}$ represents the maximum distance the foot can move within the action space; $l_{c\_\mathrm{to}\_\sup\_k}$ represents the distance from the centroid of the stable triangle formed by the current supporting feet to the base; $l_{c\_\mathrm{to}\_\mathrm{line}\_k}$ represents the distance from the centroid of the stable triangle formed by the current supporting feet to the given motion direction. $m_{1}\sim m_{8}$ are parameters related to the reward function.

(8) $$\left\{\begin{array}{c} R_{C}=-m_{0}+R_{\mathrm{foot}\;}+{\;m}_{1}R_{\mathrm{stable}}\\ R_{\mathrm{foot}\_k}=\left\{\begin{array}{c} m_{2}\;\times \;{(l_{\mathrm{foot}\_k}/l_{\mathrm{foot}\_\max})}^{m_{3}}-m_{4},\;l_{\mathrm{foot}\_k}\;\geq \;0\\ m_{5\;}-\;{m_{6}}^{l_{\mathrm{foot}\_k\;}/\;l_{\mathrm{foot}\_\max}},\;\mathrm{else} \end{array}\right.\\ R_{\mathrm{stable}\_k}=\left\{\begin{array}{c} 0\\ -m_{7}\;\times \;{(\max\{l_{c\_\mathrm{to}\_\sup\_k},{\;l}_{c\_\mathrm{to}\_\mathrm{line}\_k}\})}^{m_{8}} \end{array}\right. \end{array}\right.$$

Termination Conditions: Termination conditions are crucial for initializing the state during the training process and for early termination of erroneous actions, thereby avoiding the wastage of computational resources [21]. This paper designed the following termination conditions:

Success Termination Condition: When the robot’s base position matched the target base position, it indicated that the robot had successfully reached the destination.

Failure Termination Condition: When the robot’s base position oscillated repeatedly, or when the current swing foot position oscillated back and forth, it indicated failure of the current exploration.

Action Selection: Action selection essentially addresses the proportion problem between exploration and exploitation, i.e., a trade-off between exploration and exploitation [30]. A simple and feasible method is the ϵ-greedy method [31]. At each time step, the agent takes a random action with a fixed probability 0 < ϵ < 1, instead of greedily selecting the optimal action learned about the Q function [32]. In this paper, during greedy selection, a mask layer was added behind the output layer of the neural network, which was a list indicating whether the foot-point was feasible, to ensure that the highest scoring action was reachable, thereby avoiding a large number of unnecessary failure termination conditions. Similarly, during random selection, actions were chosen only from the list of feasible foot-points $A_{f}(s)$. Action selection is depicted as follows, where 0 ≤ ξ ≤ 1 is a uniformly random number sampled at each time step.

(9) $$\pi (s)=\left\{\begin{array}{c} \mathrm{random}\;\mathrm{action}\;\mathrm{from}\;\mathrm{feasible}\;A_{f}(s),\;\xi \;<\;\epsilon \\ \arg\;\max_{a\in A(s)}Q(s,\;a)f(s),\mathrm{otherwise} \end{array}\right.$$

Gait Planner: The gait planning strategy parameters were transformed into a neural network, consisting of two hidden layers with a size of 64 each and an activation function. In each iteration, based on the current state, the neural network outputted the Q-values for each action through a linear output layer, and then the action selection method was used to choose the action for this state.

D3QN algorithm:

The D3QN algorithm is a variant algorithm that combines aspects of both Double DQN and Dueling DQN. It aims to eliminate the maximization bias during network updates, address the issue of overestimation, and expedite algorithm convergence. Compared to the traditional DQN algorithm, D3QN primarily optimizes in two aspects. Firstly, it adopts the same optimized TD error target as the Double DQN algorithm and utilizes two networks for updates: an evaluation network (parameter $w_{e}$)) is employed to determine the next action, while a target network (parameter $w_{t}$) calculates the value of the state at time *t* + 1, thereby mitigating the problem of overestimation. The target for D3QN updates is expressed as follows, where γ represents the discount factor, $r_{t+\;1}$ denotes the reward function at time *t* + 1, and $Q(s_{t},\;a)$ denotes the state-action value function with respect to state $s_{t}$ and action $a_{t}$.

(10) $$y_{t}=\left\{\begin{array}{c} r_{t+1},\;\mathrm{if}\;\mathrm{next}\;\mathrm{state}\;\mathrm{is}\;\mathrm{final}\;\mathrm{state}\\ r_{t+1}+\gamma Q_{w_{t}}(s_{t+1},\;\arg\underset{a}{\;\max}Q_{w_{e}}(s_{t+1},a)),\;\mathrm{else} \end{array}\right.$$

Secondly, the D3QN algorithm decomposes the state–action value function into two components, consistent with the Dueling DQN algorithm, to model the state value function and the advantage function separately, thereby better handling states with smaller associations with actions. The newly defined state–action value function is represented as follows, where the maximization operation is replaced with the mean operation for enhanced stability of the algorithm. Here, $V(s_{t})$ denotes the state value function with respect to the state $s_{t}$, $A_{w_{A}}(s_{t},\;a_{t})$ represents the advantage function with respect to the state $s_{t}$ and action $a_{t}$, and *mean* denotes the mean operation.

(11) $$Q(s_{t},{\;a}_{t})=V(s_{t})+A(s_{t},\;a_{t})\;-\underset{a}{\mathrm{mean}}A(s_{t},{\;a}_{t})$$

Building upon the foundation of the D3QN algorithm, this paper established four separate MD3QN network models for each leg that incorporated a mask during action selection. The gait planning algorithm for quadrupedal robots based on the MD3QN algorithm is depicted in Algorithm 2.
**Algorithm 2** Gait Planning Based on MD3QN**Input:** learning rate *lr*, batch size *bs*, discounting factor γ, attenuation parameter ϵ, attenuation rate $\epsilon_{dec}$, minimum attenuation value $\epsilon_{min}$, target network update frequency *freq*, Soft update parameter τ, maximum number of per episode $n_{t}$, maximum number episodes $n_{s}$, maximum number of experience pool $n_{e}$**Output:** eval network that can output gaits according to state *s*Initialize the experience pool *M*, and the parameters $w_{t}$ and $w_{e}$**for** episode $e\in \{1,2,\cdots ,n_{s}\}$ **do** Reset and obtain the initialization state $s_{t}$ **for** time step $t\in \{1,2,\cdots ,n_{t}\}$ **do**  **for** foot i∈{1,2,3,4} **do**   Choose action $a_{ti}=\pi_{i}(s_{ti}|s_{ti}=s_{t})$   Compute reward $r_{ti}$   Obtain the new state $s_{t\{i+1\}}$ and done flag done   Store experience {$s_{t},a_{ti},r_{ti},s_{t\{i+1\}},done\}$ into *M*   **if** len(*M*) ≥ *bs* **then**    Sample batch data from the experience pool *M*    Obtain the target values $y_{t}$    Do a gradient descent step with loss ${\left\|y_{t}-Q_{w_{e}}(s_{ti},a_{ti})\right\|}^{2}$    update $w_{e}$   **end if**
   **if** t % freq=0
    Update $w_{t}$:$\;w_{t}\leftarrow \tau w_{e}+(1-\tau )w_{t}$   **end if**
   $s_{t}\leftarrow s_{t\{i+1\}}$
  **end for**
 **end for**
**end for**

## 5. Experiment

### 5.1. Experimental Setup

**Figure 9:**
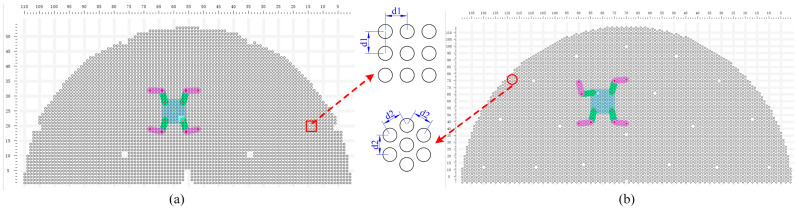
The SG tube plate environment and the tube hole distribution. (**a**) Square tube plate; (**b**) triangular tube plate.

The experiments were conducted on two types of tube plates, as illustrated in Figure 9. The algorithm’s hyperparameters are shown in Table 2, utilizing the PyTorch framework with the Adam optimizer. The training iterations $n_{s}$ and reward function parameters $m_{0}$ and $m_{2}$ were correlated with the robot’s maximum step length $l_{foot\_max}$, as outlined in Table 3. Moreover, ${\;k}_{\max}$ represents the number of foot-points that satisfied all possible inverse kinematics conditions, which was determined by the size of the robot and the hole spacing.

**Figure 10:**
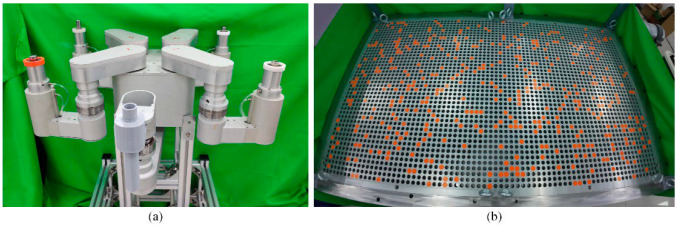
The robot HIT_Spibot and the real experimental tube plate. (**a**) HIT_Spibot. (**b**) The square-distributed tube plate. Due to the large size of the tube plate, the tube plate was taken with a wide-angle camera, which looks like an irregular curved tube plate but is actually a rectangular tube plate. The orange circles are randomly added obstructed tube holes.

Our independently developed tube plate quadruped inspection robot HIT_Spibot are depicted in Figure 10, and the parameters are outlined in Table 4. The experimental tube plate was arranged in a square distribution format, with dimensions of 43 rows by 73 columns.

To validate the feasibility and effectiveness of the proposed algorithm, three experiments were designed as follows:

Ablation experiments: Randomly selected combinations of environments and tasks were used to train four mutually influencing networks separately with MD3QN and D3QN algorithms. In each experimental group, the task action space, state space, $n_{s}$, $m_{0}$, $m_{2}$, and $l_{\mathrm{foot}\_\max}$ were set according to the dimensions of the robot and the tube plate.

Performance experiments: Obstacles were randomly added to the above environments, and the starting points and destinations of the paths were changed along the specified path direction. The trained models from the previous experiments were utilized for analysis of the adaptability of the proposed algorithm to different environments and tasks.

Practical Experiment: Obstacles were added to an actual square small tube plate, two motion paths were set, and the trained model was used to test and analyze the performance of the proposed algorithm when applied to a real robot.

### 5.2. Ablation Experiment

To evaluate the proposed method, we applied the MD3QN algorithm and the D3QN algorithm to gait motion planning. Trainings were conducted under the same environmental and hyperparameter conditions to compare algorithm performance. We introduced obstacles in both square and triangular plate environments, setting up five tasks randomly to obtain 10 different environments and tasks. Three metrics were employed to measure the planning performance, including Reward (total score of the planning results; higher is better), Step (total number of steps in the planning results; lower is better), and Stable Reward (stability score of the planning results, with stability score always non-positive; higher is better).

The final training results for the 10 environment-task combinations are presented in Table 5, where ‘S’ denotes the square plate and ‘T’ denotes the triangular plate, followed by the task number, and ‘*’ indicates the better result of the two algorithms. It can be observed that, across different environments and tasks, compared to the D3QN algorithm, the final planning results obtained using the MD3QN algorithm exhibited higher total scores, with an average increase of 6.27%; fewer steps, with an average reduction of 39.04%; and better stability, with an average increase of 58.02%. The comparative experimental results validate the effectiveness of the MD3QN algorithm.

**Figure 11:**
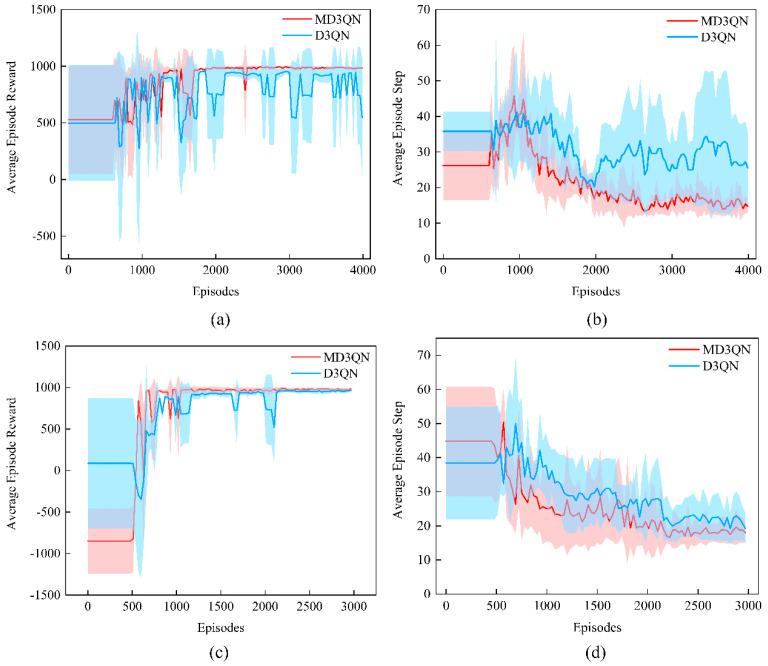
Average Episode Reward and Step during the training. (**a**) Average Episode Reward of S-1. (**b**) Average Episode Step of S-1. (**c**) Average Episode Reward of T-1. (**d**) Average Episode Step of T-1.

Figure 11 illustrates the training results for Square-1 and Triangle-1. Each curve represents the mean and variance of training scores over a specified number of rounds using five random seeds for an environment-task combination. Due to the higher exploration of ineffective routes by the D3QN algorithm in the early exploration phase, the likelihood of finding the optimal route within a limited number of rounds was lower, resulting in poor convergence and lower final scores. Under the same exploration conditions, the MD3QN algorithm could learn a control strategy with higher stable scores, as well as better convergence effectiveness.

### 5.3. Performance Experiment

**Figure 12:**
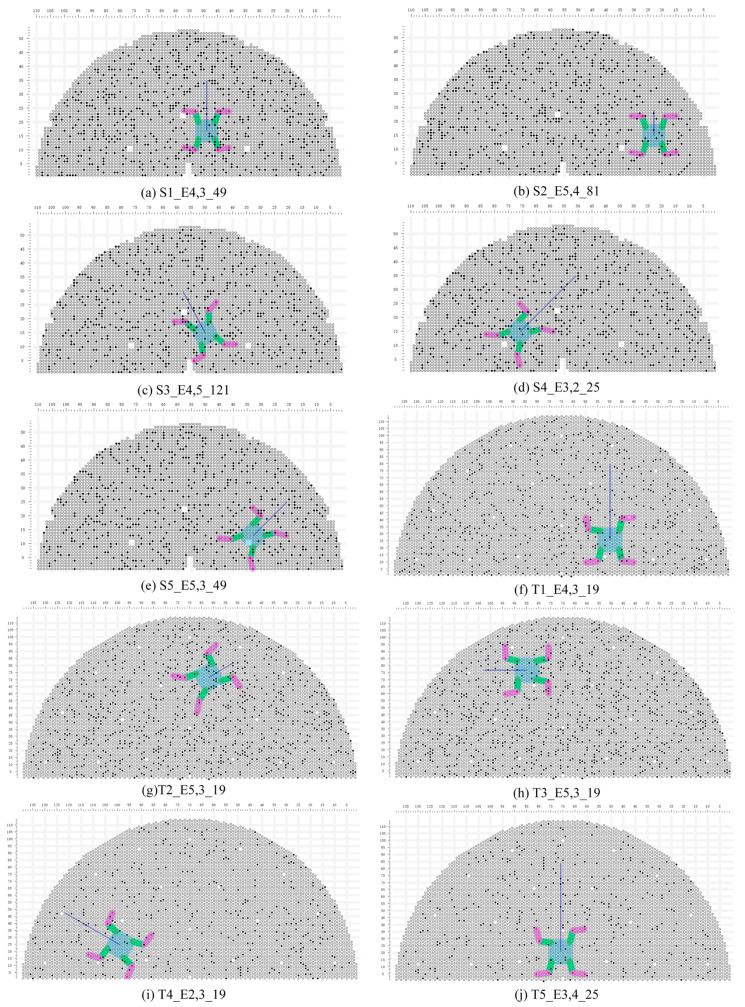
The task and environment of the performance environment. S1_E4, 5_121 denote task 1 and obstacles environment 4 under the square tube plate, the maximum step $l_{\mathrm{foot}\_\max}=5$, and the action space s $k_{\max}=121$.

Using the models trained in Section 4.4, five sets of experiments were conducted by randomly adding obstacles to the environment. We used four metrics to assess the performance of the planning: Average Success Rate (ASR, the probability of successfully reaching the goal without encountering obstacles; higher is better), Average Episode Reward (AER, the average total planning score of the five-model experiments; higher is better), Average Episode Step (AES, the average total step difference of the five-model experiments; lower is better), and Average Episode Stable Reward (AESR, the average stable score of the five-model experiments, constantly non-positive; higher is better). Figure 12 illustrates one random obstacle environment and task among the 10 sets of models for S1–T5.

The experimental results for the square and triangular boards are presented in Table 6, where—indicates that the robot failed to reach the goal in this experiment, and there was no relevant evaluation index. Across various environments, compared to the D3QN algorithm model, the MD3QN algorithm model achieved a success rate of 100%, higher total scores, fewer total steps, and higher stability scores in all environments. In other words, its performance surpassed that of the D3QN model in all aspects. The MD3QN algorithm model demonstrated superior adaptability to paths of different lengths, different start points and destinations, and randomly distributed obstructed tube holes. For both types of tube plates, when the robot navigated through narrow areas near the edges of SG (e.g., T3−E5, S2−E5), or when the robot’s walking task paths were longer (e.g., S4−E3, T5−E3), the planning models learned by MD3QN could successfully avoid obstructed holes. The performance of the MD3QN algorithm across different environments exhibited excellence and stability.

### 5.4. Practical Experiment

**Figure 13:**
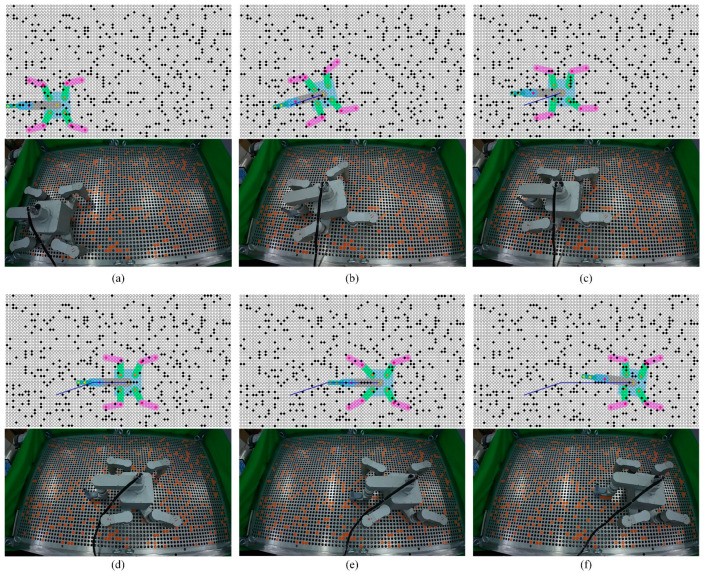
Simulation and practical comparison of the robot’s movement on the tube plate. (**a**) The initial state. (**b**) The robot reaches the end of the first segment of the path. (**c**) The robot turns. (**d**,**e**) The robot moves along the second segment of the path. (**f**) The robot reaches the target.

The motion of the robot was planned using Python, and the underlying robot controller was written in C++ to jointly control the actual motion of the robot on the tube plate. On the square-distributed tube plate, 542 obstacles were randomly added. On each defined path, the robot’s motion was planned first using the base turning planner, and then the base motion planner and the gait motion planner. The robot’s movement is depicted in Figure 13, where the black solid circles in the simulation diagram and the orange solid circles in the actual diagram represent the obstacle tube holes, and the blue line segment in the simulation diagram represents the movement path of the robot base.

The HIT_Spibot successfully executed the commands of the planning algorithm proposed in this paper. Following the motion sequence, the robot smoothly and steadily moved each leg and foot to the planned positions, then the toe modules secured to the tube plate, releasing the next swinging foot to continue the motion. Ultimately, the robot walked along the given path, reaching the target safely and successfully.

## 6. Conclusions

This paper proposed a motion planning method based on the MD3QN algorithm, which was primarily divided into the base motion planner, base turning planner, and gait motion planner. Firstly, we utilized envelope methods to calculate the workspace of the robot’s foot and base, and we employed the Lagrange multiplier method to solve the base trajectory. Secondly, based on the designed base turning criteria, we established an evaluation system using the TOPSIS method to measure the performance of the base turning planning. Thirdly, we modeled the gait motion planning of the robot as a Markov decision process and utilized the designed MD3QN algorithm to generate the optimal gait motion strategy. The MD3QN algorithm reduced the exploration of unreachable spaces by introducing a Mask layer and designing a discrete action space, thereby enhancing the convergence speed of the policy. It achieved the maximum reward value after training. The MD3QN algorithm could adapt to tube plates with different distributions and randomly obstructed tube holes, enabling the robot to smoothly traverse the given path from the initial point to the destination. Our proposed method shows promising applications in the field of nuclear power. Future research will focus on task-based robot motion planning, such as how to plan the robot base positions and the maintenance sequence of each task under multi-group area inspection tasks, so as to achieve the goal of autonomous completion of inspection tasks by the robot. And the motion algorithms proposed in this paper will provide theoretical support for future task planning motion. 

## Figures and Tables

**Table 1:** The DH table of HIT_Spibot.

Link *i*	Link Length	Joint Twist Angle	Joint Distance	Joint Angle
1	$\sqrt{b^{2}+{\;w}^{2}}$	0	$d_{1}$	$\theta_{1i}$
2	$a_{1}$	0	$d_{2}$	$\theta_{2i}$
3	$a_{2}$	0	$d_{3}$	$\theta_{3i}$
4	0	0	$d_{4i}$	0

**Table 2:** The hyperparameters of the D3QN algorithm.

Parameter	lr	bs	γ	freq	$\epsilon_{dec}$	$\epsilon_{min}$	$n_{t}$	$n_{e}$	τ	$m_{1}$	$m_{3}$	$m_{4}$	$m_{5}$	$m_{6}$	$m_{7}$	$m_{8}$
**Value**	1 × 10^−4^	64	0.99	50	2 × 10^−4^	0.05	150	10^6^	0.05	0.6	1.3	0.5	0.5	2	1.2	1.1

**Table 3:** The parameters determined by the environment and task.

$l_{foot\_max}$	$n_{s}$	$m_{0}$	$m_{2}$
1, 2	3000	−1	2
3, 4	3500	−1.5	3
5, 6	4000	−2	3.5
7, 8	5000	−3	4.5

**Table 4:** Robot structure size and motion limitation.

Parameter	b	w	$a_{1}$	$a_{2}$	$\theta_{2i},\theta_{3i}$
**Value**	130	130	130	130	[−π,π]

**Table 5:** The training results of the MD3QN and D3QN algorithms.

E-T	Reward		Step		Stable Reward	
	MD3QN	D3QN	MD3QN	D3QN	MD3QN	D3QN
S-1	1004.03 *	982.02	13 *	17	−15.10 *	−44.95
S-2	977.54 *	914.51	13 *	21	−21.17 *	−67.55
S-3	986.61 *	951.84	10 *	17	−23.96 *	−36.66
S-4	993.84 *	965.29	14 *	22	−27.34 *	−50.67
S-5	1002.91 *	902.81	14 *	34	−18.99 *	−118.67
T-1	1004.89 *	934.58	13 *	25	−21.86 *	−86.863
T-2	977.22 *	882.36	18 *	36	−55.84 *	−156.64
T-3	946.35 *	929.30	31 *	33	−82.23 *	−109.07
T-4	1007.53 *	959.55	17 *	25	−27.45 *	−74.69
T-5	965.71 *	871.77	17 *	39	−43.69 *	−93.72

* indicates the better result of the two algorithm.

**Table 6:** The performance experiment results of the MD3QN and D3QN algorithms.

E-T	Metrics	E1		E2		E3		E4		E5	
		MD3QN	D3QN	MD3QN	D3QN	MD3QN	D3QN	MD3QN	D3QN	MD3QN	D3QN
S-1	ASR	100%	100%	100%	100%	100%	100%	100% *	0%	100%	100%
	AER	984.02 *	971.53	995.09 *	940.93	969.76 *	954.34	920.56 *	—	1002.5 *	990.43
	AES	18 *	21	20*	30	29 *	30	33 *	—	12 *	17
	AESR	−48.05 *	−56.15	−37.24 *	−104.85	−71.42 *	−101.67	−96.10 *	—	−15.12 *	−31.84
S-2	ASR	100%	100%	100%	100%	100%	100%	100%	100%	100% *	0%
	AER	977.54 *	858.84	963.18 *	913.56	941.15 *	845.13	858.79 *	745.11	970.70 *	—
	AES	13 *	34	14*	24	27 *	40	46 *	54	13 *	—
	AESR	−21.17 *	−67.55	−36.14 *	−65.76	−68.38 *	−153.26	−117.93 *	−247.79	−35.06 *	—
S-3	ASR	100%	100%	100%	100%	100% *	0%	100%	100%	100%	100%
	AER	941.96 *	931.67	919.98 *	918.90	933.56 *	—	858.00 *	608.24	946.08 *	935.84
	AES	19 *	20	19 *	24	21 *	—	40 *	82	17 *	19
	AESR	−55.509 *	−57.67	−72.54 *	−78.14	−68.72 *	—	−135.01 *	−322.58	−39.23 *	−60.69
S-4	ASR	100% *	0%	100%	100%	100%	100%	100%	100%	100% *	0%
	AER	995.52 *	—	986.30 *	874.38	944.49 *	566.69	973.55 *	757.92	969.32 *	—
	AES	13 *	—	22 *	44	62 *	139	28 *	76	28 *	—
	AESR	−26.39 *	—	−50.67 *	−164.50	−139.56 *	−585.64	−60.94 *	−308.23	−70.51 *	—
S-5	ASR	100%	100%	100%	100%	100% *	0%	100% *	0%	100%	100%
	AER	989.34 *	874.92	994.63 *	633.28	881.29 *	—	915.64 *	—	964.36 *	660.56
	AES	12 *	41	22 *	92	67 *	—	55 *	—	32 *	93
	AESR	−37.97 *	−146.84	−52.99 *	−454.45	−247.05 *	—	−194.46 *	—	−105.93 *	−436.95
T-1	ASR	100%	100%	100%	100%	100% *	0%	100%	100%	100%	100%
	AER	1006.39 *	915.66	1007.55 *	983.56	1003.95 *	-	993.08 *	890.05	986.48 *	973.93
	AES	21 *	36	17 *	25	25 *	-	37 *	6	9 *	12
	AESR	−34.15 *	−124.38	−27.41 *	−49.89	−40.70 *	-	−94.05 *	−190.03	−27.61 *	−45.92
T-2	ASR	100%	100%	100%	100%	100%	100%	100%	100%	100%	100%
	AER	976.20 *	882.36	952.88 *	771.43	934.07 *	702.06	982.77 *	901.42	832.58 *	820.29
	AES	18 *	36	24 *	61	33 *	81	9 *	29	57*	60
	AESR	−56.84 *	−156.64	−84.36 *	−288.60	−118.52 *	−367.11	−29.12 *	−133.83	−204.70 *	−219.68
T-3	ASR	100%	100%	100%	100%	100%	100%	100%	100%	100%	100%
	AER	943.30 *	911.81	869.51 *	850.48	821.34 *	770.12	872.00 *	844.32	895.76 *	892.82
	AES	30 *	38	54 *	56	83 *	81	57 *	59	45 *	45
	AESR	−87.83 *	−123.91	−185.78 *	−208.67	−250.32 *	−332.46	−183.42 *	−226.92	−150.42 *	−153.60
T-4	ASR	100%	100%	100% *	0%	100% *	0%	100% *	0%	100%	100%
	AER	973.07 *	948.82	1005.14 *	—	609.85 *	—	842.27 *	—	834.7 *	573.73
	AES	21 *	25	33 *	—	138 *	—	64 *	—	94 *	146
	AESR	−65.12 *	−83.55	−56.45 *	—	−552.60 *	—	−233.17 *	—	−277.48 *	−606.02
T-5	ASR	100% *	0%	100%	100%	100%	100%	100% *	0%	100%	100%
	AER	962.06 *	—	846.25 *	712.74	895.50 *	422.69	711.85 *	—	957.11 *	770.64
	AES	21 *	—	37 *	67	40 *	131	73 *	—	16 *	50
	AESR	37.26 *	—	−151.82 *	−204.49	−134.45 *	−481.98	−254.75 *	—	−44.53 *	188.02

* indicates the better result of the two algorithm.

## Data Availability

The data are contained within the article.

## References

[1] Ou Y., Xu B., Cai H., Zhao J., Fan J. An overview on mobile manipulator in nuclear applications. Proceedings of the 2022 IEEE International Conference on Real-Time Computing and Robotics (RCAR).

[2] Obrutsky L. (2017). Nuclear steam generator tube inspection tools. Steam Generators for Nuclear Power Plants.

[3] Joseph S., Sakthivel S., Jose J., Jagadishan D., Visweswaran P., Murugan S., Amarendra G., Bhaduri A. (2019). Prototype fast breeder reactor steam generator inspection system for tube inspections. Machines, Mechanism and Robotics: Proceedings of iNaCoMM.

[4] Xu B., Li G., Zhang K., Cai H., Zhao J., Fan J. Design and motion performance of new inspection robot for steam generator heat transfer tubes. Proceedings of the 2021 IEEE International Conference on Real-Time Computing and Robotics.

[5] Zhang K., Fan J., Xu T., Liu Y., Xing Z., Xu B., Zhao J. (2024). Dimensional synthesis of an Inspection Robot for SG tube-sheet. Nucl. Eng. Technol..

[6] Tsounis V., Alge M., Lee J., Farshidian F., Hutter M. (2020). DeepGait: Planning and Control of Quadrupedal Gaits Using Deep Reinforcement Learning. IEEE Robot. Autom. Lett..

[7] Alexander W. (2018). Optimization-Based Motion Planning for Legged Robots. Ph.D. Thesis.

[8] Alexander W., Bellicoso C., Hutter M., Buchli J. (2018). Gait and trajectory optimization for legged systems through phase-based endeffector parameterization. IEEE Robot. Autom. Lett..

[9] Zhang Z., Yan J., Xin K., Zhai G., Liu Y. (2021). Efficient Motion Planning Based on Kinodynamic Model for Quadruped Robots Following Persons in Confined Spaces. IEEE/ASME Trans. Mechatron..

[10] Ha S., Xu P., Tan Z., Levine S., Tan J. (2002). Learning to walk in the real world with minimal human effort. arXiv.

[11] Ladosz P., Weng L., Kim M., Oh H. (2022). Exploration in deep reinforcement learning: A survey. Inf. Fusion.

[12] Zhang W., Tan W., Li Y. (2020). Locmotion control of quadruped robot based on deep reinforcement learning: Review and prospect. J. Shandong Univ. (Health Sci.).

[13] Richard S., Andrew G. (1998). Reinforcement learning: An introduction. IEEE Trans. Neural Netw..

[14] Jiang N., Deng Y., Nallanathan A. Deep Reinforcement Learning for Discrete and Continuous Massive Access Control optimization. Proceedings of the ICC 2020—2020 IEEE International Conference on Communications.

[15] Ibarz J., Tan J., Finn C., Kalakrishnan M., Pastor P., Levine S. (2021). How to train your robot with deep reinforcement learning: Lessons we have learned. Int. J. Robot. Res..

[16] Mnih V., Kavukcuoglu K., Silver D., Graves A., Antonoglou I., Wierstra D., Riedmiller M. (2013). Playing Atari with Deep Reinforcement Learning. Comput. Sci..

[17] Lillicrap T., Hunt J., Pritzel A., Heess N., Erez T., Tassa Y., Silver D., Wierstra D. (2015). Continuous control with deep reinforcement learning. arXiv.

[18] Wang X., Fu H., Deng G., Liu C., Tang K., Chen C. (2023). Hierarchical Free Gait Motion Planning for Hexapod Robots Using Deep Reinforcement Learning. IEEE Trans. Ind. Inform..

[19] Wang Z., Schaul T., Hessel M., Hasselt H., Lanctot M., Freitas N. Dueling Network architectures for deep reinforcement learning. Proceedings of the 33rd International Conference on International Conference on Machine Learning.

[20] Jain D., Iscen A., Caluwaerts K. Hierarchical Reinforcement Learning for Quadruped Locomotion. Proceedings of the IEEE/RSJ International Conference on Intelligent Robots and Systems.

[21] Peng X., Abbeel P., Levine S., Panne M. (2018). DeepMimic: Example-Guided Deep Reinforcement Learning of Physics-Based Character Skills. Trans. Graph..

[22] Li J., Wang J., Yang S., Zhou K., Tang H. (2016). Gait Planning and Stability Control of a Quadruped Robot. Computational Intelligence and Neuroscience.

[23] Liu C., Chen Q., Wang D. (2011). CPG-inspired workspace trajectory generation and adaptive locomotion control for quadruped robots. IEEE Trans. Syst. Man Cybern. Part B (Cybern.).

[24] Sun Y., Pancheri F., Rehekampff C., Lueth T. (2024). TurBot: A Turtle-Inspired Quadruped Robot Using Topology Optimized Soft-Rigid Hybrid Legs. IEEE/ASME Trans. Mechatron..

[25] Kim J., Alspach A., Yamane K. Snapbot: A reconfigurable legged robot. Proceedings of the 2017 IEEE/RSJ International Conference on Intelligent Robots and Systems (IROS).

[26] Bertsekas D.P. (2014). Constrained Optimization and Lagrange Multiplier Methods.

[27] Olson D. (2004). Comparison of weights in TOPSIS models. Math. Comput. Model..

[28] Karimi M., Yusop Z., Yusop S. (2010). Location decision for foreign direct investment in ASEAN countries: A TOPSIS approach. Int. Res. J. Financ. Econ..

[29] Liu W., Li B., Hou L., Xu Y. (2022). Review of Quadruped Robot Research Based on Deep Reinforcement Learning. J. Qilu Univ. Technol..

[30] Sutton R., Barto A. (2018). Reinforcement Learning: An Introduction.

[31] Watkins C. (1989). Learning from Delayed Rewards. Ph.D. Thesis.

[32] Tokic M., Palm G. (2011). Value-Difference Based Exploration: Adaptive Control between Epsilon-Greedy and Softmax. Deutsche Jahrestagung für Künstliche Intelligenz.

